# ASO Author Reflections: The PlasmaJet^®^ Device Contributes to an Increase in the Number of Complete Cytoreductive Surgeries for Ovarian Cancer Patients

**DOI:** 10.1245/s10434-022-11827-3

**Published:** 2022-04-28

**Authors:** G. M. Nieuwenhuyzen-de Boer, H. J. van Beekhuizen

**Affiliations:** 1 Department of Gynecologic Oncology, Erasmus MC Cancer Institute, University Medical Center, Rotterdam, The Netherlands; 2 Department of Obstetrics and Gynecology, Albert Schweitzer Hospital, Dordrecht, The Netherlands

## Past

The most important independent prognostic factor for survival of patients with advanced-stage epithelial ovarian cancer (EOC) is the completeness of cytoreductive surgery.^1^ Standard surgical treatment of advanced-stage ovarian carcinoma with electrosurgery cannot always result in complete cytoreductive surgery (CRS), especially when many small metastases are found on the mesentery and intestinal surfaces.

Available literature showed that the use of the PlasmaJet^®^ device contributes to an increase in the number of complete cytoreductive surgeries for ovarian cancer patients.^2^ A randomized controlled study (RCT) to demonstrate safety and effectiveness was lacking.^3^ For this reason, the authors investigated whether adjuvant use of a neutral argon plasma device helps to increase the complete cytoreduction rate without an increase in the number of complications.^4^

## Present

The PlaComOv Study on the effectiveness and safety of the PlasmaJet device during CRS shows that surgery with the adjuvant use of the PlasmaJet is associated with a significant higher percentage of complete CRS procedures for patients with resectable disease.^5^ This benefit was even stronger in the subset analysis of patients with peritoneal carcinomatosis, defined as 50 or more metastatic lesions on the peritoneum, diaphragm, or mesentery.

In the intervention group, the PlasmaJet was used 104 times during surgery (75%). In 41% of the procedures, the gynecologic oncologists gave their opinion that the PlasmaJet was necessary (12%) or very useful (29%) to achieve complete CRS. The PlasmaJet simplifies the removal of lesions at locations of the diaphragm without muscle contractions, peritoneum, or superficial tumor lesions at vulnerable sites such as the mesentery and the colon.

The surgical complication rate did not differ significantly between the two groups. A relaparotomy was performed for eight patients in the intervention group. No relaparotomy was related to the use of the PlasmaJet.

## Future

Considering that the surgical outcome has an important impact on both progression-free survival (PFS) and overall survival (OS),^1^ the authors recommend that clinicians consider using the PlasmaJet during CRS. Still, survival data need to mature for assessment of the effect on PFS and OS outcomes.

The patients in the intervention group reported more favorable health scores than the patients in the control group. A possible explanation could be the lower percentage of colostomies in the intervention group (9 vs 20) or the long-term protective effect of the PlasmaJet, resulting in less tissue damage. Research into the difference in quality of life is in progress.

The mean total health care costs for use of the PlasmaJet in CRS for ovarian cancer will be higher than for conventional CRS. A cost analysis of treatment with the adjuvant use of the PlasmaJet is under construction. A cost-effectiveness analysis can be performed once data about quality of life and survival are available.
